# Dataset of results from numerical simulations of increased storm intensity in an estuarine salt marsh system

**DOI:** 10.1016/j.dib.2021.107336

**Published:** 2021-08-28

**Authors:** Natascia Pannozzo, Nicoletta Leonardi, Iacopo Carnacina, Rachel Smedley

**Affiliations:** a Department of Geography and Planning, School of Environmental Sciences, University of Liverpool, Chatham Street, Liverpool L69 7ZT, United Kingdom; b Department of Civil Engineering, School of Civil Engineering and Built Environment, Liverpool John Moores University, Byrom Street, Liverpool L3 3AF, United Kingdom

**Keywords:** Salt marshes, Storm surges, Delft3D

## Abstract

This article contains data outlining the effects of increased storm intensity on estuarine salt marshes, previously evaluated in Pannozzo et al. (2021), using the Ribble Estuary, in North West England, as a case study. The hydrodynamic model Delft3D was used to simulate various surge height scenarios and evaluate the effects of increasing surge height on the sediment budget of the system. The data shows that an increase in storm intensity (i.e. surge height) promotes flood dominance and triggers a net import of sediment, positively contributing to the sediment budget of the marsh platform and the estuarine system. The timing of the storm surge relative to high or low tide, the duration of the surge and the presence of vegetation do not cause major changes in the sediment budget. This dataset could be used to evaluate how increased storm intensity might influence the sediment budget of estuaries in comparison to other types of coastal systems (e.g., bays) to illustrate how the response of salt marshes to increased storm intensity varies with a change in the hydrodynamics and sediment delivery dynamics of the system.

**Specifications Table**

**Table:** 

Subject	Geosciences
Specific subject area	Coastal hydrodynamics
Type of data	Table, figure
How data were acquired	Numerical simulations, Delft3D modelling framework
Data format	Analysed data
Parameters for data collection	Gaussian functions were added to the water level time-series at the offshore boundary of the domain.
Description of data collection	The model was forced for a month with water level time-series at the offshore boundary and water discharge time-series at the landward boundary.
Data source location	University of Liverpool, Liverpool, United Kingdom
Data accessibility	https://doi.org/10.5281/zenodo.4511397
Related research article	N. Pannozzo, N. Leonardi, I. Carnacina, R. Smedley, Salt marsh resilience to sea-level rise and increased storm intensity, Geomorphology, 389, 107,825. https://doi.org/10.1016/j.geomorph.2021.107825

## Value of the Data

• The modelled storm surge scenarios can be used to evaluate how increased storm intensity might influence the sediment budget of salt marshes and estuaries.
• This dataset can be used by other coastal scientists or engineers to make a comparison with other types of coastal systems (e. g. bays) to illustrate how the response of salt marshes to increased storm intensity varies with a change in the hydrodynamics and sediment delivery dynamics of the system.
• Data could be used to guide an investigation into sediment delivery dynamics of real storm surges occurred in the estuary.

## 1 Data Description

**Fig. 1:**
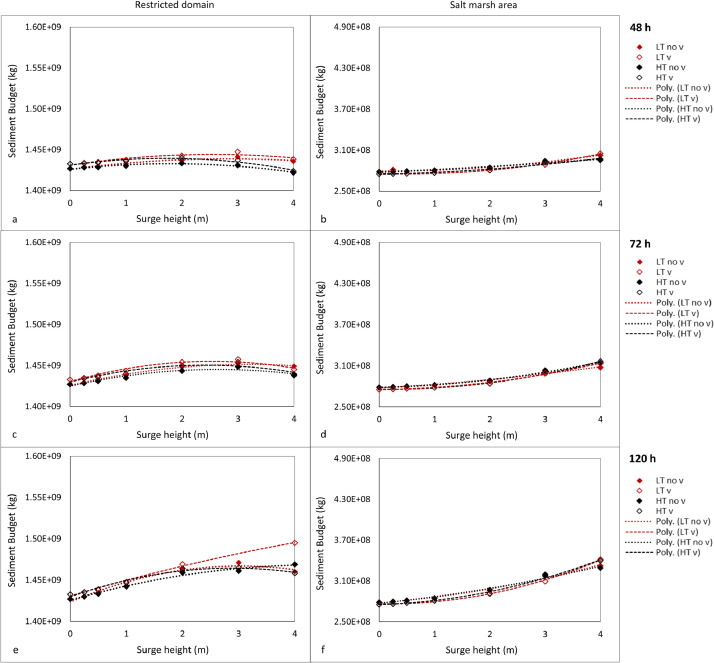
Sediment budget integrated across the entire area of the restricted domain (a, c, e) and the saltmarsh (b, d, f) for each surge height, for surges occurring at high tide (HT) and low tide (LT) without vegetation (no v) and with vegetation (v), for surges of different durations occurring at neap tide; (see Figure 3 in [4] for surges occurring at spring tide); scenarios run using an ideal only-mud bed composition.

**Fig. 2:**
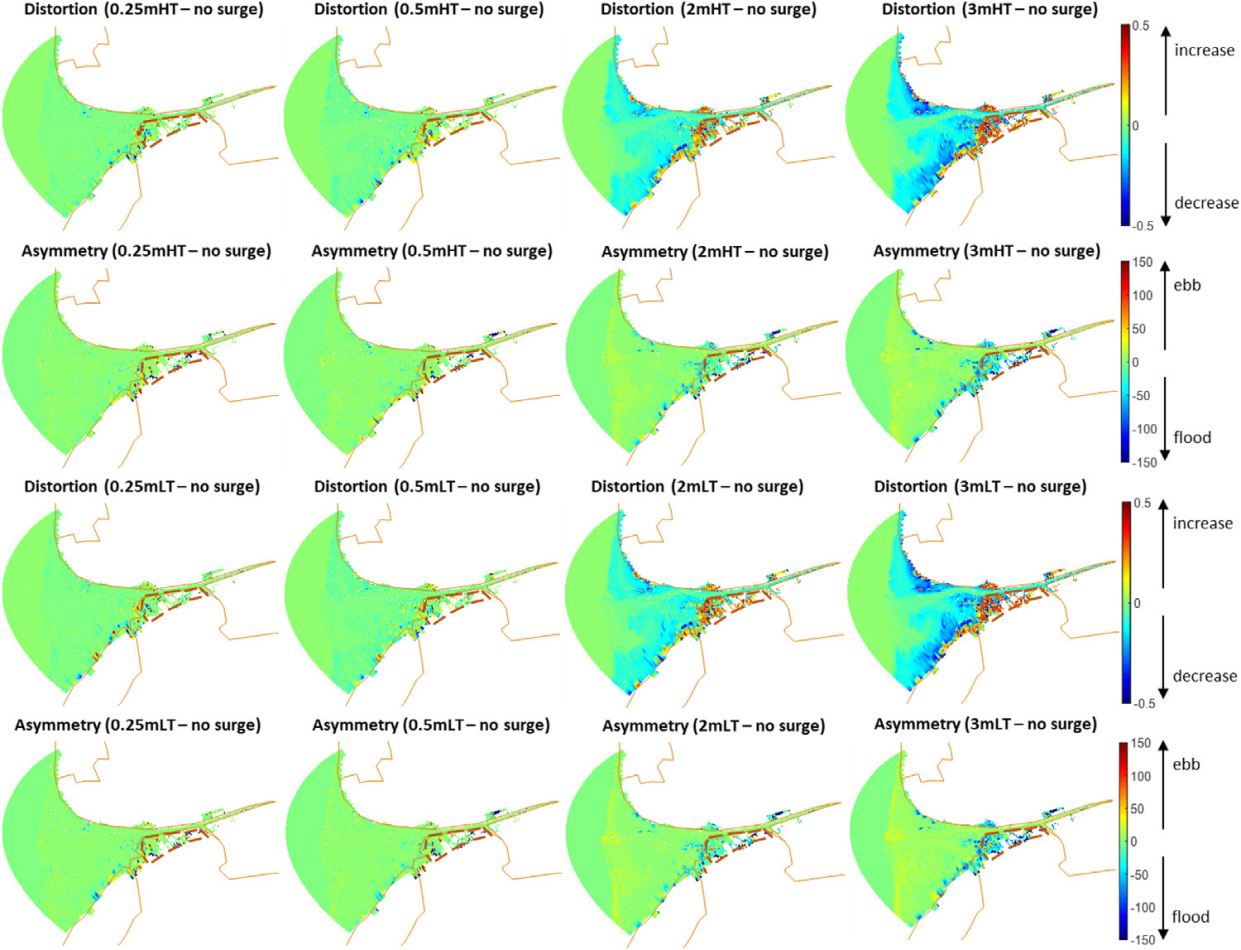
Difference between tidal distortion (A_4–2_) and asymmetry (Δθ) of 0.25 m, 0.5 m, 2 m and 3 m surge scenarios and the no surge scenario at current sea-level (see Figure 7 from [4] for the 1 m and 4 m scenarios). When Δθ is positive there is an increase in ebb dominance with respect to the no surge scenario, when it is negative there is an increase in flood dominance; when A_4–2_ is positive, the degree of the asymmetry is more significant, vice versa when it is negative. The continuous brown lines correspond to the land boundary. The area enclosed by the brown dashed lines is the salt marsh.

**Fig. 3:**
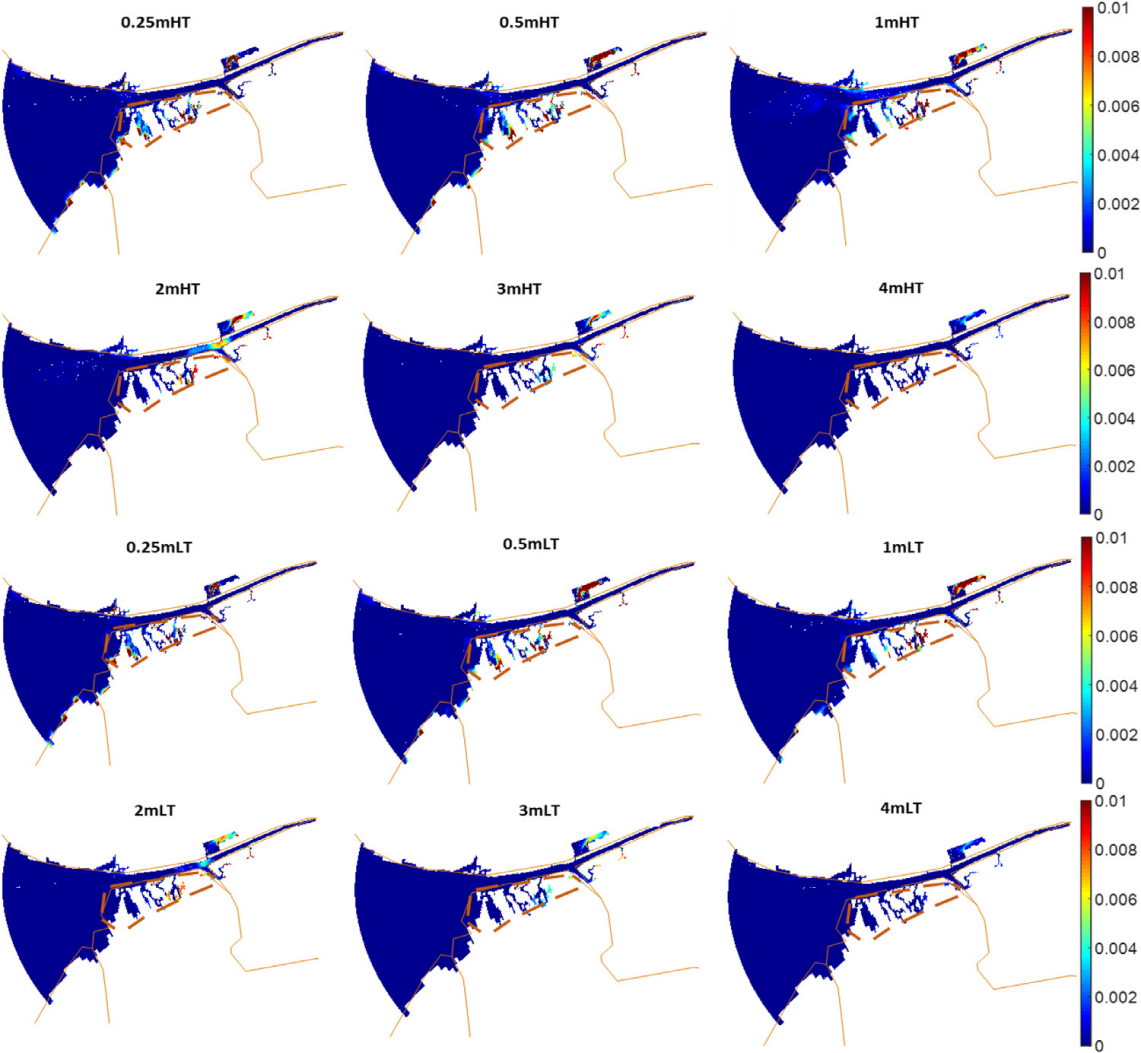
Difference between bottom friction in all surge scenarios and the no surge scenario during flood phase. Calculation of bottom friction followed [7]. The continuous brown lines correspond to the land boundary. The area enclosed by the brown dashed lines is the salt marsh.

**Fig. 4:**
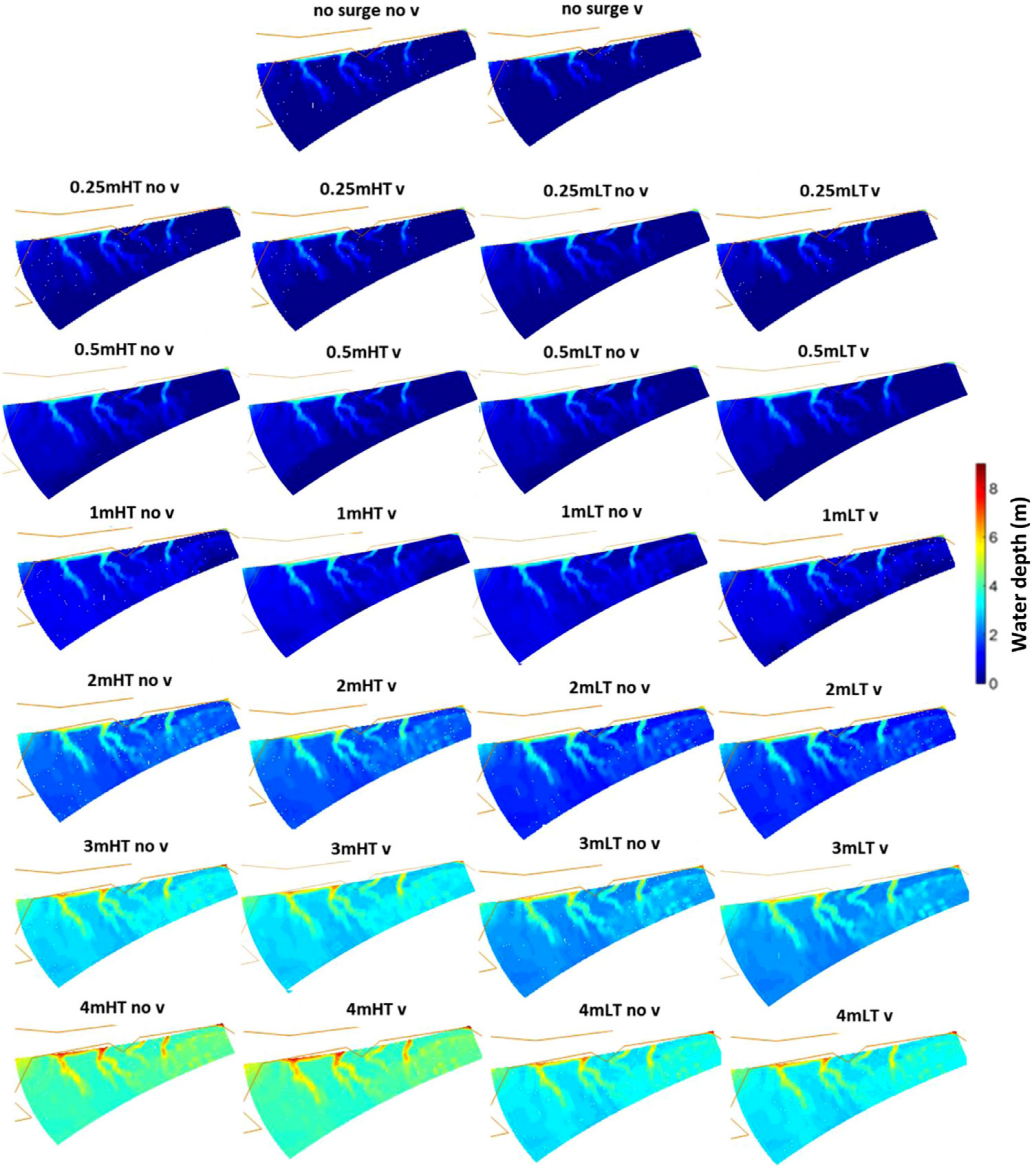
Water depth on the salt marsh platform during flood phase for vegetated and hypothetical non-vegetated scenarios for all surge scenarios. The continuous brown lines correspond to the land boundary.

Scenarios of increased storm intensity modelled for the Ribble Estuary, North-West England, are presented here. Numerical simulations representing different storm surge heights were conducted using the numerical finite-difference model Delft3D [1]. The main details of the numerical grid used for the Ribble estuary can be found in [2,3] and [4]. Each scenario was simulated for a month from 1st January until 31st January 2008 by varying the timing of occurrence of the surge with respect to high or low tide, the tidal range, the duration of the surge and the presence of vegetation. The occurrence of the surge was simulated using a gaussian function that was added to the initial offshore boundary water level time-series. The various intensities were modelled by simulating different surge heights: 0 m, 0.25 m, 0.5 m, 1.0 m, 2.0 m, 3.0 m and 4.0 m. These values were selected at regular intervals within the range observed by an extreme value analysis of storm surge residuals along the UK coastline (Table 1). These scenarios were repeated using different typical mid-latitude durations of 48 h (σ = 6 h), 72 h (σ = 9 h) and 120 h (σ = 15 h), for surges occurring at spring tide and neap tide and for surges peaking at high tide and low tide, with and without the presence of vegetation. The sediment budget at the end of each simulation was calculated for the marsh platform and for a restricted area of the estuarine system (restricted domain) to describe the amount of accretion (positive values) or erosion (negative values) for each scenario [5]. Fig. 1 shows that, for the neap tide scenarios, the sediment budget of both marsh platform and restricted domain increases with an increase in surge height, with no visible alterations between surges peaking at high tide and surges peaking at low tide and between surges of different durations. Tidal analysis (Fig. 2) indicates that an increase in surge height causes a shift towards a more flood dominated system, which is responsible for an increase in sediment import and explains the increase in sediment budget showed by Fig. 1. Fig. 3 shows that an increase in surge height causes a nearshore reduction in friction, which explains the shift towards flood dominance indicated by Fig. 2. The presence of vegetation does not cause major changes to the sediment budget; however, on the marsh platform, it is responsible for a lower sedimentation at lower surge heights and higher sedimentation at higher surge heights (Fig. 1). Fig. 4 shows that the water overflows the creeks only for higher water depths, while it stays constrained in the creeks at lower water depths, explaining the variations in sedimentation caused by the vegetation. Overall, data shows that storm surges positively contribute to the resilience of salt marshes and estuarine systems.

**Table 1:** Exceedance probabilities (p) of storm surge heights along the UK shoreline with return period (RP) of 2 years, 10 years, 25 years, 50 years, 100 years and 500 years. Tidal level records from 1952 to 2015 have been downloaded from British Oceanographic data centre and residuals have been fitted using a generalized extreme values distribution to obtain the heights in the table [6].

Station Name	*p* = 0.5(RP=2)	*p* = 0.1(RP=10)	*p* = 0.04(RP=25)	*p* = 0.02(RP=50)	*p* = 0.01(RP=100)	*p* = 0.002(RP=500)
ABERDEEN	0.88	1.11	1.18	1.22	1.25	1.3
AVONMOUTH	1.78	2.37	2.6	2.75	2.89	3.14
BANGOR	1	1.27	1.33	1.36	1.39	1.42
BARMOUTH	1.39	2.05	2.36	2.58	2.8	3.27
BOURNEMOUTH	0.81	1.01	1.08	1.13	1.17	1.25
CROMER	1.53	2.05	2.22	2.32	2.4	2.54
DEVONPORT	0.78	0.93	0.98	1	1.02	1.06
DOVER	1.23	1.55	1.68	1.76	1.84	1.99
FELIXSTOWE	1.58	2.18	2.42	2.59	2.73	3.02
FISHGUARD	0.81	1.06	1.18	1.26	1.35	1.54
HARWICH	1.46	1.88	2.04	2.15	2.24	2.4
HEYSHAM	1.74	2.28	2.45	2.54	2.62	2.74
HINKLEY	1.51	1.95	2.12	2.23	2.33	2.51
HOLYHEAD	0.97	1.24	1.34	1.4	1.44	1.52
ILFRACOMBE	1.07	1.24	1.28	1.29	1.3	1.31
ISLAY	1.07	1.35	1.45	1.52	1.58	1.68
JERSEY	1.07	1.31	1.39	1.44	1.48	1.54
KINLOCHBERVIE	1.06	1.43	1.6	1.73	1.84	2.09
LEITH	1.06	1.35	1.42	1.46	1.5	1.54
LERWICK	0.57	0.71	0.77	0.81	0.84	0.9
LIVERPOOL	1.76	2.24	2.37	2.44	2.5	2.57
LOWESTOFT	1.52	2.04	2.25	2.4	2.53	2.78
MILFORD HAVEN	0.91	1.19	1.3	1.36	1.42	1.54
MILLPORT	1.35	1.66	1.78	1.86	1.93	2.07
MORAY FIRTH	0.83	1.26	1.61	1.96	2.41	4.01
MUMBLES	1.1	1.56	1.78	1.94	2.1	2.45
NEWLYN	0.69	0.88	0.96	1.02	1.07	1.19
NEWHAVEN	0.87	1.05	1.12	1.16	1.2	1.27
NEWPORT	1.74	2.29	2.58	2.79	3	3.51
PORTPATRICK	1.11	1.44	1.54	1.59	1.63	1.69
PORTRUSH	1.06	1.21	1.24	1.25	1.26	1.26
PORTSMOUTH	0.84	1.08	1.23	1.34	1.47	1.79
SHEERNESS	1.75	2.4	2.63	2.78	2.9	3.11
ST MARY'S	0.6	0.77	0.83	0.87	0.91	0.98
STORNOWAY	0.84	1.07	1.15	1.21	1.26	1.35
TOBERMORY	1.21	1.45	1.5	1.52	1.53	1.54
ULLAPOOL	0.92	1.44	1.79	2.08	2.43	3.42
WEYMOUTH	0.78	0.94	0.99	1.03	1.05	1.1
WHITBY	1.2	1.8	2.2	2.56	2.98	4.2
WICK	0.8	1.01	1.09	1.14	1.18	1.25
WORKINGTON	1.54	1.9	2.05	2.15	2.24	2.42

## 2 Experimental Design, Materials and Methods

The data was collected using the FLOW module of the numerical finite-difference model Delft3D, which computes non-steady flow and transport phenomena implementing Navier-Stokes and transport equations [1]. The suspended load is calculated through the advection–diffusion equation and the bed-load transport through the Van Rijn formulation [8]. These formulations are applied to multiple cohesive and non-cohesive sediment fractions. The upward diffusion and sediment dropping related to the settling velocities are evaluated to compute the exchange of non-cohesive sediments between the bed and the flow near the bottom [8]. The erosion and deposition of cohesive sediments are computed through the Partheniades–Krone formulations [9]. The model was constrained for a month within two open boundaries, one 20 km offshore forced with water level time-series and one across the River Ribble forced with discharge time-series. Details about the set-up of the boundary conditions can be found in Table 1 and Fig. 2 from [4]. To simulate the effects of storm surges, gaussian functions were added to the initial offshore boundary water level time-series. The vegetation presence on the marsh platform and its effect on the flow field were computed following the formulation of [10], which models plant stems as rigid cylinders. This allows the model to compute the three-dimensional effect of vegetation on drag and turbulence, by accounting for an extra source term of friction force in the momentum equation and for an extra source term of turbulent kinetic energy dissipation in the k-e equations, both generated by the cylindrical plant structures. The MATLAB package T-TIDE [11] was employed to analyse the effects of storm surges on the tidal signal.

## CRediT Author Statement

**Natascia Pannozzo:** Conceptualisation, Methodology, Data production and analysis, Data curation, Writing – original draft preparation; **Nicoletta Leonardi:** Conceptualisation, Methodology, Data production and analysis, Supervision, Writing – reviewing and editing; **Iacopo Carnacina:** methodology, supervision, Writing – reviewing & editing; **Rachel Smedley:** Conceptualisation, Supervision, Writing – reviewing & editing.

## Declaration of Competing Interest

The authors declare that they have no known competing financial interests or personal relationships that could have appeared to influence the work reported in this paper.
